# Supplementary material for: Task-residual effective connectivity of motor network in transient ischemic attack
Source: Commun Biol. 2023 Aug 14;6:843. doi: 10.1038/s42003-023-05212-3 (PMC10425379; doi:10.1038/s42003-023-05212-3)
Supplement: Supplementary file 2 — Supplementary Information [file 42003_2023_5212_MOESM2_ESM.pdf]

Supplementary Information

Task-Residual Effective Connectivity of Motor Network in Transient Ischemic Attack

Truc Chu[1,2,†], Seonjin Lee[1,2,†], Il-Young Jung[3,†], Youngkyu Song[4], Hyun Ah Kim[5], Jong Wook Shin[6,*] , Sungho Tak[1,2,*]

[1]Research Center for Bioconvergence Analysis, Korea Basic Science Institute, Cheongju 28119, Republic of Korea
[2]Graduate School of Analytical Science and Technology, Chungnam National University, Daejeon 34134, Republic of Korea
[3]Department of Rehabilitation Medicine, Chungnam National University Sejong Hospital, Sejong 30099, Republic of Korea
[4]Bio-Chemical Analysis Team, Korea Basic Science Institute, Cheongju 28119, Republic of Korea
[5]Department of Rehabilitation Medicine, Chungnam National University Hospital, Daejeon 35015, Republic of Korea
[6]Department of Neurology, Chungnam National University Sejong Hospital, Sejong 30099, Republic of Korea

† These authors contributed equally

* Corresponding authors, email addresses: dr.shin@hanmail.net (J.W. Shin), stak@kbsi.re.kr (S. Tak)

Supplementary Note 1: Correlation Analysis between Effective Connectivity and ABCD^2 Score

To investigate the stroke risk relevance of effective connectivity, a Pearson correlation analysis was performed to calculate the relationship between DCM parameters that showed significant group differences and ABCD^2 scores. ABCD^2 is a prognosis score for stroke risk after TIA, where a higher ABCD^2 score indicates that the patient with TIA has a higher risk of stroke[1].

Statistically significant associations between variables ($p < 0.05$) are illustrated in Supplementary Fig. 1. During right hand movement, as the connectivity strength from the left PMC to the ipsilateral right M1 was increased, the ABCD^2 score was increased (r = 0.53, p = 0.042). A higher ABCD^2 score indicates a higher risk of stroke after TIA[1]. On the other hand, the decrease in connectivity to the SMA was significantly correlated with the increase in ABCD^2 score during both right hand (from the right IPL to the SMA: r = −0.6, p = 0.019) and left hand movement (from the right M1 to the SMA: r = −0.59, p = 0.021).

In the studies of the neural phenotypes of mental illness, motivation or behavioral engagement can influence the degree of brain activation during task performance, which would hinder inferences of the association between task engagement and brain activations (i.e., less brain activation in patients could be concomitant with altered behavior performance)[2]. To overcome this, Zhang et al.[2] proposed removing the task-related activities from the time series to obtain the task-residual BOLD activity[3], which could facilitate the identification of behavioral/motivation-independent in task-related processes between patients and HCs. In this study we also removed the task-related effects from the BOLD signal (task-residual) to adjust for behavioral effects in the responses before performing DCM. Nonetheless, the underlying effective connectivity was still different between the patient with TIA and the HC group and showed the significant relationship with the risk factor of stroke, ABCD^2 score. A previous study showed that patients with TIA with greater disinhibition in the motor cortex showed higher stroke risk (higher

$ABCD^2$ scores)[4]. In line with this finding, our results further verified the relationship between stroke risk and the aberrant effective connectivity in patients with TIA. The higher alteration in connectivity in the patients (increased connectivity to the ipsilateral M1 or decreased connectivity to the SMA) reflected the higher severity and risk of stroke after TIA onset.

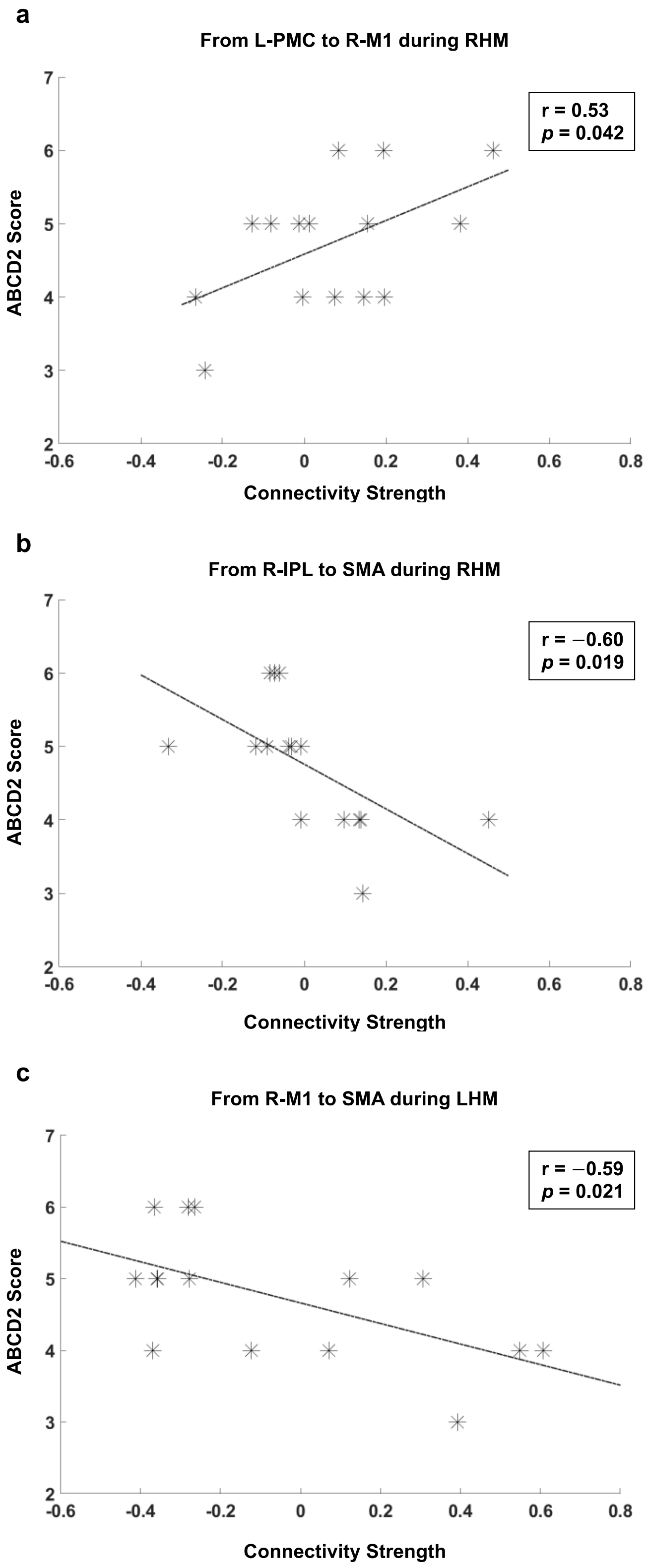

Supplementary Fig. 1 Correlation between task-residual effective connectivity and ABCD^2 scores of patients with transient ischemic attack (TIA). Associations of ABCD^2 scores with

effective connectivity (a) from the left premotor cortex to right primary motor cortex during right hand movement (RHM), (b) from the right inferior parietal lobule (IPL) to supplementary motor area (SMA) during RHM, and (c) from the right primary motor cortex (M1) to SMA during left hand movement (LHM). Asterisk represents experimental results of each individual having a pair of ABCD[2] score and connectivity strength. For the numerical source data, see Supplementary Data 4.

Supplementary References

1. Johnston, S. C. *et al.* Validation and refinement of scores to predict very early stroke risk after transient ischaemic attack. *Lancet* **369**, 283–292 (2007).

2. Zhang, S. & Li, C.-S. R. A neural measure of behavioral engagement: task-residual low-frequency blood oxygenation level-dependent activity in the precuneus. *Neuroimage* **49**, 1911–1918 (2010).

3. Fair, D. A. *et al.* A method for using blocked and event-related fMRI data to study “resting state” functional connectivity. *Neuroimage* **35**, 396–405 (2007).

4. Edwards, J. D. *et al.* Changes in intracortical excitability after transient ischemic attack are associated with ABCD2 score. *Stroke* **42**, 728–733 (2011).
