# Supplementary material for: Task-residual effective connectivity of motor network in transient ischemic attack
Source: Commun Biol. 2023 Aug 14;6:843. doi: 10.1038/s42003-023-05212-3 (PMC10425379; doi:10.1038/s42003-023-05212-3)
Supplement: Supplementary file 3 — Description of Additional Supplementary Files [file 42003_2023_5212_MOESM3_ESM.pdf]

Description of Additional Supplementary Files

File Name: Supplementary Data 1

Description: The source data behind Fig. 1 and Fig. 2. The individual data of each connectivity during right and left hand movements. Abbreviations: HC, healthy controls; TIA, transient ischemic attack; SMA, supplementary motor area; PMC, premotor cortex; M1, primary motor cortex; IPL, inferior parietal lobule.

File Name: Supplementary Data 2

Description: The source data behind Fig. 3. The individual data of each connectivity of the external control regions of interests during both right and left and movement. Abbreviations: HC, healthy controls; TIA, transient ischemic attack; mPMC, medial prefrontal cortex; PREC, precuneus; IPC, inferior parietal cortex

File Name: Supplementary Data 3

Description: The source data behind Fig. 4. The individual data of each connectivity during right and left hand movements for Model 1 (fully connected model) and Model 2 (model without bidirectional connectivity to the SMA). Abbreviations: HC, healthy controls; TIA, transient ischemic attack; SMA, supplementary motor area; PMC, premotor cortex; M1, primary motor cortex; IPL, inferior parietal lobule.

File Name: Supplementary Data 4

Description: The source data behind Supplementary Fig. 1. The individual data of ABCD^2 scores and the correlated effective connectivity. Abbreviations: TIA, transient ischemic attack; SMA, supplementary motor area; PMC, premotor cortex; M1, primary motor cortex; IPL, inferior parietal lobule.
