# Supplementary material for: Task-residual effective connectivity of motor network in transient ischemic attack
Source: Commun Biol. 2023 Aug 14;6:843. doi: 10.1038/s42003-023-05212-3 (PMC10425379; doi:10.1038/s42003-023-05212-3)
Supplement: Supplementary file 8 — Reporting Summary [file 42003_2023_5212_MOESM8_ESM.pdf]

Corresponding author(s): Sungho Tak
Last updated by author(s): Aug 1, 2023

Reporting Summary

Nature Portfolio wishes to improve the reproducibility of the work that we publish. This form provides structure for consistency and transparency in reporting. For further information on Nature Portfolio policies, see our Editorial Policies and the Editorial Policy Checklist.

Statistics

For all statistical analyses, confirm that the following items are present in the figure legend, table legend, main text, or Methods section.

n/a	Confirmed	
☐	☒	The exact sample size (*n*) for each experimental group/condition, given as a discrete number and unit of measurement
☐	☒	A statement on whether measurements were taken from distinct samples or whether the same sample was measured repeatedly
☐	☒	The statistical test(s) used AND whether they are one- or two-sided<br>*Only common tests should be described solely by name; describe more complex techniques in the Methods section.*
☐	☒	A description of all covariates tested
☐	☒	A description of any assumptions or corrections, such as tests of normality and adjustment for multiple comparisons
☐	☒	A full description of the statistical parameters including central tendency (e.g. means) or other basic estimates (e.g. regression coefficient) AND variation (e.g. standard deviation) or associated estimates of uncertainty (e.g. confidence intervals)
☐	☒	For null hypothesis testing, the test statistic (e.g. F, t, r) with confidence intervals, effect sizes, degrees of freedom and P value noted<br>*Give P values as exact values whenever suitable.*
☐	☒	For Bayesian analysis, information on the choice of priors and Markov chain Monte Carlo settings
☐	☒	For hierarchical and complex designs, identification of the appropriate level for tests and full reporting of outcomes
☐	☒	Estimates of effect sizes (e.g. Cohen's *d*, Pearson's *r*), indicating how they were calculated

Our web collection on statistics for biologists contains articles on many of the points above.

Software and code

Policy information about availability of computer code

Data collection	The MR application software included in the commercial MRI scanner (7 Tesla Philips Achieva) was used to collect the data in this study.
Data analysis	We used open source software SPM12 (version 7771) and DCM12.5 that were developed at the Wellcome Centre for Human Neuroimaging, UK (https://www.fil.ion.ucl.ac.uk/spm/), and xjView toolbox developed by Dr. Xu Cui at the Stanford University (https://www.alivelearn.net/xjview/about/) to analyse the data in this study.

For manuscripts utilizing custom algorithms or software that are central to the research but not yet described in published literature, software must be made available to editors and reviewers. We strongly encourage code deposition in a community repository (e.g. GitHub). See the Nature Portfolio guidelines for submitting code & software for further information.

Data

Policy information about availability of data

All manuscripts must include a data availability statement. This statement should provide the following information, where applicable:

- Accession codes, unique identifiers, or web links for publicly available datasets
- A description of any restrictions on data availability
- For clinical datasets or third party data, please ensure that the statement adheres to our policy

The data generated during and/or analyzed during this study are available from the corresponding author upon request. Numerical source data for Figures 1 and 2 are provided in Supplementary Data 1. Numerical source data for Figure 3 and 4 are stored in Supplementary Data 2 and 3, respectively.

Human research participants

Policy information about studies involving human research participants and Sex and Gender in Research.

Reporting on sex and gender	The findings of this study apply to sex. Sex was only considered in this study design, and sex was determined based on self-reporting. This study involved 11 male and 4 female patents with transient ischemic attack, and 8 male and 20 female healthy adults. Sex-based analysis was performed (i.e., covariate of sex ratio for each group was included in the general linear model and dynamic causal modelling analyses.)
Population characteristics	Healthy adults participated in this study (mean age = 63.8 years, standard deviation = 6.6 years). The healthy participants had no history of neurological diseases within the previous five years.<br>Patients with transient ischemic attack participated in this observational study (mean age = 61.5 years, standard deviation = 9.5 years). The patients with TIA were diagnosed by experienced clinical neurologists within 24 hours after symptom onset which was defined as the point at which the patient reported no longer being in a normal condition. All patients were scanned using MRI within a month after being diagnosed with TIA (mean = 16.2 days, standard deviation = 7.4 days).
Recruitment	All participants were recruited from national university hospitals near the study location as well as through local advertising. Specifically, patients with transient ischemic attack were diagnosed and recruited by experienced clinical neurologists at the Department of Neurology, Chungnam National University Sejong Hospital. Age-matched healthy adults were also recruited and screened by a medical doctor at the Department of Rehabilitation Medicine, Chungnam National University Sejong Hospital. All participants were informed in detail about the nature of the study and provided written informed consent for the study. We confirmed that all methods were carried out in accordance with the relevant institutional guidelines and regulations.<br>There was a sex-ratio bias between healthy adults and patients with transient ischemic attack who participated in this study. In order to reduce the effects of this bias on results, we included the sex difference as a nuisance covariate in the general linear model and dynamic causal modelling analyses. Also, a previous cohort study on transient ischemic attack reported that stroke recurrence was not significantly different between male and female participants (Amy et al., 2019). Therefore, based on the standard analysis method and previous findings, the effect of sex-ratio bias is not likely to significantly impact the results and findings of our study. We addressed this issue in the discussion section of manuscript in detail.<br><br>Amy, Y.X., Penn, A.M., Lesperance, M.L., Croteau, N.S., Balshaw, R.F., Votova, K., Bibok, M.B., Penn, M., Saly, V., Hegedus, J. and Zerna, C., 2019. Sex differences in presentation and outcome after an acute transient or minor neurologic event. JAMA neurology, 76(8), pp.962-968.
Ethics oversight	The research was approved by the institutional review board of the Chungnam National University Hospital (2019-07-004-001) on 7 August 2019.

Note that full information on the approval of the study protocol must also be provided in the manuscript.

Field-specific reporting

Please select the one below that is the best fit for your research. If you are not sure, read the appropriate sections before making your selection.

☒ Life sciences ☐ Behavioural & social sciences ☐ Ecological, evolutionary & environmental sciences

For a reference copy of the document with all sections, see nature.com/documents/nr-reporting-summary-flat.pdf

Life sciences study design

All studies must disclose on these points even when the disclosure is negative.

Sample size	We determined sample sizes of participants (healthy adults = 28, patients with transient ischemic attack = 15) based on statistical power analysis results (Desmond and Glover, 2002). Neuroimaging studies have reported the sample size estimates (required to achieve 80% power at a 5% level of significance) in the range of 12 to 25 subjects (Desmond and Glover, 2002; Goulden et al., 2012; Torrisi et al., 2018). In terms of DCM-fMRI study, using the power analysis based on 1.5T fMRI data, Goulden et al., (2012) showed that 20 subjects per group are required to achieve 80% power at a significance level of 0.05. This minimum number of samples can be further reduced by increasing the effect size via ultra-high field (e.g., 7T) MRI system (van der Zwaag et al., 2009; Hale et al., 2010; Tak et al., 2018). Using these gains at 7T in the effect size, Torrisi et al., (2018) performed power analysis between magnetic field strength and showed that fewer subjects at 7T are necessary to produce comparable effects at lower field strength 3T. In this study, we estimated the DCM connectivity strengths from the 7T fMRI images. Therefore, considering (i) the increase in effect sizes at 7T and (ii) power analysis results of previous neuroimaging studies, the sample sizes used in this study fall within the range of the sample sizes to ensure 80% power at a 5% level of significance. We also performed simulation-based power analysis using DCM connectivity data where experimental task (Rehme et al., 2011), ROIs of connectivity model, and participants (patients with stroke) were similar with our study. This resulted in a required sample size of 14.22 for each group to achieve 80% power at a 5% significance level, which confirmed the sample size used in this study met the criterion for achieving sufficient statistical power.<br><br>- Desmond, J.E. and Glover, G.H., 2002. Estimating sample size in functional MRI (fMRI) neuroimaging studies: statistical power analyses. -Journal of Neuroscience Methods, 118(2), pp.115-128.<br>- Goulden, N., Elliott, R., Suckling, J., Williams, S.R., Deakin, J.F., McKie, S., 2012. Sample size estimation for comparing parameters using dynamic causal modeling. Brain Connect. 2, 80–90.

- Torrisi, S., Chen, G., Glen, D., Bandettini, P.A., Baker, C.I., Reynolds, R., Yen-Ting Liu, J., Leshin, J., Balderston, N., Grillon, C., Ernst, M., 2018. Statistical power comparisons at 3T and 7T with a go / nogo task. NeuroImage 175, 100–110.
- van der Zwaag, W., Francis, S., Head, K., Peters, A., Gowland, P., Morris, P., Bowtell, R., 2009. fMRI at 1.5, 3 and 7 T: Characterising Bold signal changes. NeuroImage 47, 1425–1434.
- Tak, S., Noh, J., Cheong, C., Zeidman, P., Razi, A., Penny, W.D., Friston, K.J., 2018. A validation of dynamic causal modelling for 7T fMRI. J. Neurosci. Methods 305, 36–45.
- Hale, J.R., Brookes, M.J., Hall, E.L., Zumer, J.M., Stevenson, C.M., Francis, S.T., Morris, P.G., 2010. Comparison of functional connectivity in default mode and sensorimotor networks at 3 and 7T. Magn. Reson. Mater. Phy., 23, 339-349.
- Rehme, A. K., Eickhoff, S. B., Wang, L. E., Fink, G. R., Grefkes, C., 2011. Dynamic causal modeling of cortical activity from the acute to the chronic stage after stroke. Neuroimage 55 (3), 1147–1158.

Data exclusions

No data were excluded from the analyses.

Replication

All findings reported in this study were statistically significant. A threshold based on the Bayesian criterion of posterior probability of p > 0.99 was used to verify strong evidence (statistical significance) of experimental findings. To improve the reproducibility of our findings, we thoroughly described research methodology and used standard experimental protocol and analysis methods. We also provided the numerical source data - individual connectivity strengths - used for estimating group-level connectivity strengths. The data and codes used during this study are available from the corresponding author upon request.

Randomization

This study lies in an observational study. We observed groups of healthy adults and patients with transient ischemic attack to learn about the potential difference of connectivity among brain regions between two groups. Participants did not receive any interventions such as drugs relevant to this study.
We recruited participants according to the selection criteria for each group. We did not subjectively exclude or allocate participants into experimental groups. Thus, as this is an observational study, further randomization is not relevant to this study.

Blinding

Blinding was not relevant to this study, because as described above this study lies in an observational study.

Reporting for specific materials, systems and methods

We require information from authors about some types of materials, experimental systems and methods used in many studies. Here, indicate whether each material, system or method listed is relevant to your study. If you are not sure if a list item applies to your research, read the appropriate section before selecting a response.

Materials & experimental systems

n/a	Involved in the study	
☒	☐	Antibodies
☒	☐	Eukaryotic cell lines
☒	☐	Palaeontology and archaeology
☒	☐	Animals and other organisms
☒	☐	Clinical data
☒	☐	Dual use research of concern

Methods

n/a	Involved in the study	
☒	☐	ChIP-seq
☒	☐	Flow cytometry
☐	☒	MRI-based neuroimaging

Magnetic resonance imaging

Experimental design

Design type

Task, block design experiment

Design specifications

The subjects were instructed to perform close and open their right (R) or left (L) hands synchronized with 1Hz flickering circle (movement cue). The experiment comprised 20 second blocks of task interspersed with 20 second rest blocks. The sequence of L, and R blocks was pseudo-randomized. The number of blocks was 9 and the number of trials for each block was 20. The total scan time was 395 seconds.

Behavioral performance measures

We did not measure the behavioral performance. However, participants were monitored by an experimenter inside the magnet room to confirm whether the participants were actually performing the visually cued fist-closing movement. We measured the ABCD2 score from patients with transient ischemic attack (TIA), to assess the risk of stroke after TIA.

Acquisition

Imaging type(s)

Functional and structure images were acquired.

Field strength

7 Tesla

Sequence & imaging parameters

Functional images were acquired using a T2*-weighted gradient echo-planar imaging sequence. Specific imaging parameters are as follows: field of view (RL/AP/FH) = 192 × 192 x 100 mm, matrix size = 96 x 96, 39 interleaved slices with 0.6 mm slice gap, slice thickness = 2 mm, slice orientation = transverse, and TE/TR/flip angle = 2500ms/22ms/80 degree.

Structural images were acquired using a three-dimensional magnetization prepared rapid gradient echo (3D MPRAGE) sequence. Specific imaging parameters are as follows: field of view (RL/AP/FH) = 234 × 234 x 188 mm, matrix size = 336 x 336, 269 slices, slice thickness = 0.7 mm, slice orientation = sagittal, and TE/TR/flip angle = 5.5ms/2.6ms/7 degree.

Area of acquisition: A whole brain scan was used.

Diffusion MRI: ☐ Used ☒ Not used

Preprocessing

Preprocessing software: Preprocessing was performed using SPM12 software - version 7771(https://www.fil.ion.ucl.ac.uk/spm/). For each subject, the data were corrected for the intensity non-uniformity of functional and structural images at 7T MRI. All the frames were spatially realigned, co-registered with high-resolution anatomical images, normalized into MNI space and smoothed with an 8mm full-width at half-maximum Gaussian kernel.

Normalization: Both functional and structural data were normalized/standardized using nonlinear registration implemented in SPM12 software. Structural image was coregistered to the mean functional image. Spatial normalization then performed via the segmentation routine - unified segmentation algorithm (Ashburner and Friston, 2005). Finally, functional volumes were spatially normalized to the Montreal Neurological Institute (MNI) space using the normalization parameters estimated during unified segmentation.

Ashburner, J. and Friston, K.J., 2005. Unified segmentation. Neuroimage, 26(3), pp.839-851.

Normalization template: ICBM152 template was used for normalizing the data into the MNI space.

Noise and artifact removal: Motion artifact in functional images was removed by spatially realigning all time frames to the first time frame using a least sqaures approach and a six parameter (rigid body) spatial transformation. In the following general linear analysis (GLM), parameters for the residual head movement on fMRI images were included in the design matrix of GLM as nuisance variables to mitigate the confounding influence of head motion on the inference of activated voxel location.
In order to remove the physiological noises arising from cardiac activity and respiration, we first estimated the principal component of voxels within segmented tissue masks of the white matter and cerebrospinal fluids. We then regressed out those systemic confounds from the fMRI time series.

Volume censoring: We did not performed volume censoring.

Statistical modeling & inference

Model type and settings: Mass-univariate analysis (voxel-wise GLM) was applied to fMRI data. At the first level, the GLM with the canonical hemodynamic response function was fitted to the individual fMRI signal to estimate the effects of interest for each subject. At the second-level, the population effects were estimated using the random-effects summary statistics approach.

Effect(s) tested: The conditions of right and left fist-closing movement were included as covariates of interest in the GLM analysis. ANOVA or factorial designs were not used.

Specify type of analysis: ☒ Whole brain ☐ ROI-based ☐ Both

Statistic type for inference (See Eklund et al. 2016): Voxel-wise inference was used.

Correction: FWE corrected p-value < 0.05 was used.

Models & analysis

n/a	Involved in the study
☐	☒ Functional and/or effective connectivity
☒	☐ Graph analysis
☒	☐ Multivariate modeling or predictive analysis

Functional and/or effective connectivity: Effective connectivity was estimated using dynamic causal modelling (DCM) analysis. Effective connectivity was formulated as a generative model of how measured fMRI data were caused by interactions among brain regions at the neural level. The effective connectivity parameters were then estimated by fitting the generative model to fMRI data using Bayesian inference formed as the spectral DCM.
